# Risk score for futile recanalization: integrating cerebral circulation time and collateral cascade

**DOI:** 10.3389/fnagi.2026.1737846

**Published:** 2026-04-10

**Authors:** Xiaowen Song, Guo-feng Zhao, Yong-Lin Qin, Zhi-Bin Bai, Jia-Jie Ji, Gang Deng

**Affiliations:** Center of Interventional Radiology and Vascular Surgery, Department of Radiology, Zhongda Hospital, Medical School, Southeast University, Nanjing, China

**Keywords:** acute ischemic stroke, cerebral circulation time, cerebral collateral cascade, futile recanalization, microvascular reperfusion

## Abstract

**Introduction:**

Impaired microvascular reperfusion despite successful recanalization may contribute to futile recanalization (FR) following thrombectomy. By combining cerebral circulation time (CCT) and cerebral collateral cascade (CCC) to evaluate hemodynamic and microcirculatory disturbances, we aim to develop and validate a new comprehensive prognostic model to predict FR after thrombectomy.

**Methods:**

This retrospective cohort study involved a thorough analysis of perfusion and collateral status (CCT and CCC) in consecutive acute ischemic stroke (AIS) patients presented with large vessel occlusion who underwent thrombectomy and achieved successful recanalization between January 2022 and December 2024. Independent predictors of FR were obtained from the least absolute shrinkage and selection operator regression and multivariable logistic regression. A nomogram model was constructed, and its discrimination and calibration were assessed.

**Results:**

This study included 182 patients, of whom 73 (40.11%) suffered from FR. Feature importance identified ischemic core volume (OR 1.01, 95% CI 1.00–1.03, *p* = 0.017), CCC (OR 3.09, 95% CI 1.24–8.40, *p* = 0.020), relative change of CCT (OR 5.87, 95% CI 1.45–26.36, *p* = 0.016), and neutrophil-to-lymphocyte ratio (OR 2.73, 95% CI 1.30–5.96, *p* = 0.009) as the most important predictors. The final model was discriminatory for predicting 3-month FR (area under the receiver operating characteristic curve 0.733, 95% CI 0.659–0.806) and had good calibration (Brier 0.201, 95% CI 0.175–0.226; Hosmer–Lemeshow test, *p* = 0.770). Decision curve analysis indicated a higher mean net benefit at lower treatment thresholds (up to 0.90).

**Conclusion:**

The new prognostic model provides a valuable tool for predicting FR, thereby improving individualized stroke management.

## 1 Introduction

Acute ischemic stroke (AIS) represents the predominant form of all strokes and constitutes a leading contributor to adult disability and death worldwide. Approximately half (46%) of AIS cases result from acute intracranial large vessel occlusion (LVO). Endovascular therapy (EVT), especially mechanical thrombectomy (MT), administered either independently or combined with intravenous thrombolysis (IVT), has been established as the first-line treatment for both acute anterior and posterior circulation LVO (Albers et al., 2018; Liu et al., 2020; Nogueira et al., 2018). While grade 2b–3 on the modified Thrombolysis in Cerebral Infarction (mTICI) scale is achieved, approximately half exhibit limited functional recovery (mRS 3–6 or 4–6 at 90 days) with premorbid mRS ≤ 2, a phenomenon termed futile recanalization (FR) (Meinel et al., 2022; Tian et al., 2023; Gan et al., 2026).

The Kaplan–Meier 7-year survival estimates for 3-month survivors with mRS 0–1, 2–3, and 4–5 were 67, 50, and 23%, respectively (Magalhães et al., 2014). Early prognostic stratification of long-term clinical impairment based on information available in the acute phase of AIS would be valuable for optimizing personalized stroke care, rehabilitation strategies, and patient expectations. Several clinical and imaging biomarkers associated with FR in the anterior circulation have been identified so far (Gan et al., 2026; Zhou et al., 2022; Yang et al., 2023; Huang et al., 2024; Bauerle et al., 2026), with multiple attempts to combine them into standardized prediction models (Meinel et al., 2022; Guan et al., 2023; Riou-Comte et al., 2020). However, most of these investigations were solely restricted to traditional and basic clinical and imaging factors such as age, baseline Alberta Stroke Program Early CT Score (ASPECTS), National Institutes of Health Stroke Scale (NIHSS) score, and collaterals, underestimating the no-reflow phenomenon. Sustained microvascular hypoperfusion post-recanalization persists despite macrovascular patency, driving the pathogenesis of FR (Ng et al., 2022). Given this dilemma, there is a need to introduce a comprehensive, readily accessible model that integrates traditional clinical characteristics and novel multimodal laboratory and imaging biomarkers, focusing on microcirculatory perfusion and hemodynamics, to predict FR with improved accuracy and precision.

The FR occurrence rate ranges from 32.4 to 56.7%. Proposed pathophysiological mechanisms include microvascular compromise, particularly the no-reflow phenomenon (Deng et al., 2022; Tian et al., 2023). Cerebral circulation time (CCT) is a clinically significant hemodynamic index that reflects cerebrovascular reserve capacity and dynamic microcirculatory responses to perfusion disturbances (Udoetuk et al., 2007). This microcirculation-sensitive parameter demonstrates independent prognostic value for functional outcomes in AIS-LVO patients following EVT (Wang J. Q. et al., 2022). Recently, a multidimensional assessment model integrating arterial collateral pathways, tissue-level perfusion patterns, and venous outflow dynamics was introduced, named the cerebral collateral cascade (CCC). This comprehensive approach specifically emphasizes comprehensive collateral blood flow and microperfusion status (Faizy et al., 2022), positioning CCC as a sensitive imaging biomarker for predicting FR in EVT-treated AIS-LVO patients (Huang et al., 2025; Gong et al., 2025). The role of the no-reflow phenomenon in FR has garnered increasing attention, as it is the clinical manifestation of functional and structural alterations in the microvasculature during the ischemia–reperfusion process (Jia et al., 2024). With CCT providing microcirculation perfusion and hemodynamic information and CCC being a surrogate marker of microvascular integrity, although the combined role of CCC and CCT in FR prediction after EVT has been barely investigated, it could be fairly promising.

Consequently, we postulate that integrating CCT and CCC may provide a peri-procedural biomarker for comprehensive microcirculatory assessment and enable accurate prediction of FR after MT, thereby better informing shared decision-making and improving personalized treatment strategies, including intensive monitoring or adjuvant treatment.

## 2 Materials and methods

### 2.1 Study population

We performed a retrospective cohort study of consecutive AIS-LVO patients undergoing EVT at a comprehensive stroke center (Zhongda Hospital, Southeast University) between January 2022 and December 2024. Subjects were retrospectively identified from the clinical database at the center. Final inclusion in the study cohort required documented successful recanalization, defined as a mTICI grade of 2b or 3. Patients were excluded for any of the following: (1) prestroke functional dependence (mRS score ≥ 2), (2) intra-arterial thrombolysis, and (3) unqualified digital subtraction angiography (DSA), including poor quality or incomplete DSA images.

Data were gathered at baseline by trained research staff following a standard protocol, including baseline epidemiological and clinical characteristics [sex, age, NIHSS score on admission, onset to reperfusion time (ORT), concomitant treatment with intravenous thrombolysis (IVT), vascular risk factors (history of stroke, hypertension, diabetes, atrial fibrillation, and coronary heart disease)] as well as interventional angiographic characteristics [groin puncture to recanalization time (PRT), extent of recanalization as assessed by the treating neurointerventionalist with the mTICI score]. Stroke severity was assessed on admission, with NIHSS ≥15 considered severe stroke. Hemorrhagic transformation (HT) was found by computed tomography (CT) after EVT. Blood samples were collected on admission preceding EVT procedures. Cutoff values for these blood biomarkers were determined using reference intervals predetermined by the Laboratory of Zhongda Hospital. Laboratory parameters included lymphocyte count, neutrophil count, monocyte count, fasting blood glucose, hypersensitive C-reactive protein (HS-CRP), fibrinogen (Fbg), and creatine. Inflammatory profiles are represented by neutrophil-to-lymphocyte ratio (NLR) derived by dividing absolute neutrophil count by absolute lymphocyte count (Gong et al., 2021) and the systemic inflammatory response index (SIRI) calculated by the formula: absolute neutrophil count × absolute monocyte count/absolute lymphocyte count (Huang et al., 2024).

### 2.2 Imaging analysis

Upon arrival at the emergency department of our stroke center, patients with highly suspected AIS-LVO undergo non-contrast head CT, CT angiography (CTA), and CT perfusion (CTP) immediately for evaluation, followed by expedited transfer to the angiographic suite.

ASPECTS was determined from pre-treatment head non-contrast CT images. The baseline ischemic core was automatically delineated as tissue exhibiting relative cerebral blood flow (rCBF) < 30% compared to contralateral normal regions. Hypoperfused tissue volume was defined as areas with Tmax delays >6 s. The mismatch volume (difference between hypoperfused tissue and ischemic core) was considered penumbra. All perfusion images were generated algorithmically from the acquired CTP data.

**Figure 1:**
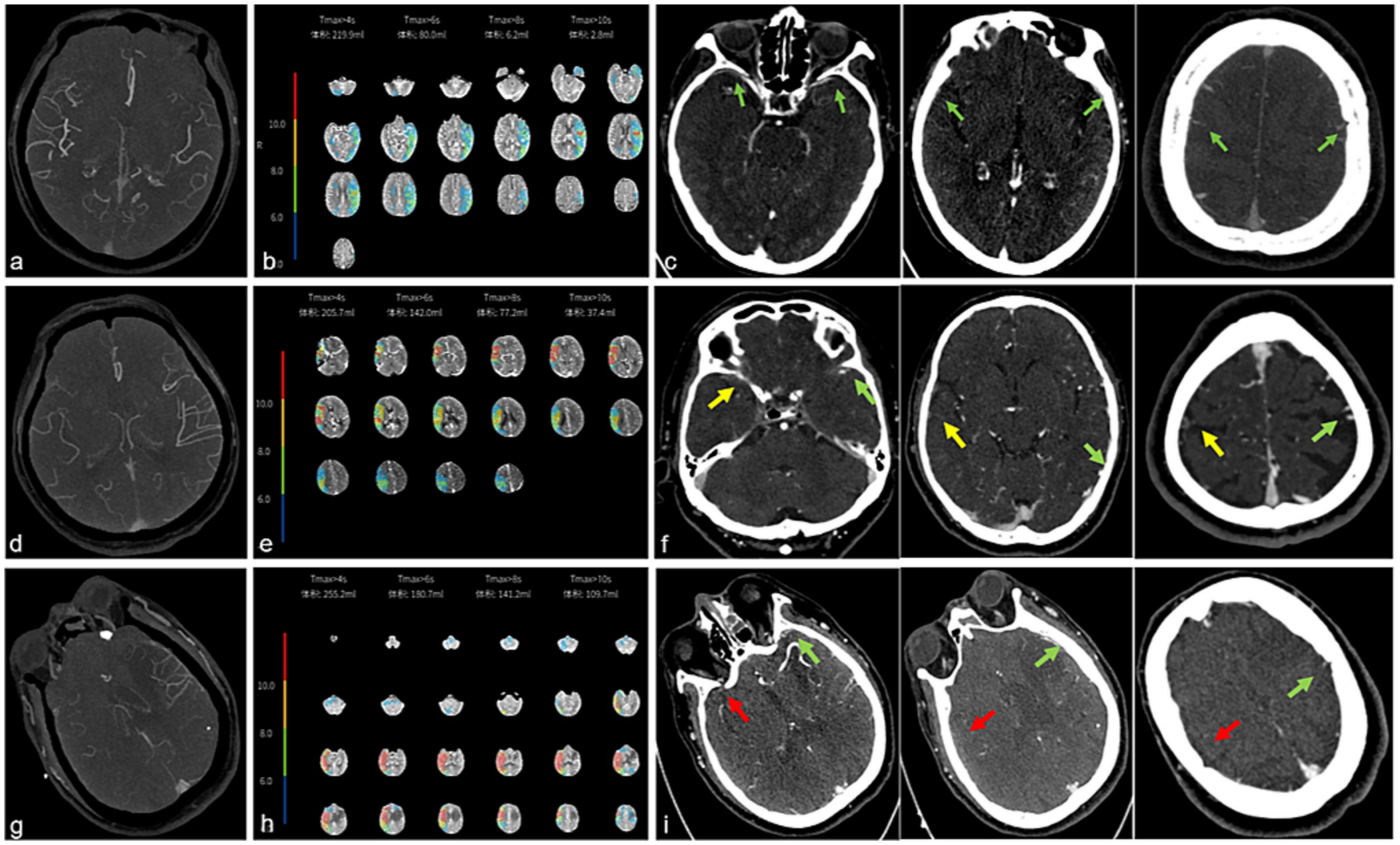
Images demonstrating the cerebral collateral cascade (CCC) profile. The first patient presented with moderate pial arterial collaterals (Tan scale = 2) **(a)**, favorable tissue-level collaterals (HIR = 0.035) **(b)**, and a robust venous outflow profile (COVES = 6) **(c)** on the infarction left hemisphere. The second patient presented with limited pial arterial collaterals (Tan scale = 1) **(d)**, favorable tissue-level collaterals (HIR = 0.26) **(e)**, and a moderate venous outflow profile (COVES = 3) **(f)** on the infarction right hemisphere. The third patient presented with poor pial arterial collaterals (Tan scale = 0) **(g)**, poor tissue-level collaterals (HIR = 0.61) **(h)**, and a poor venous outflow profile (COVES = 0) **(i)** on the infarction left hemisphere. Green arrows display robust venous outflow with grade 2, yellow arrows indicate poor venous outflow with grade 1, and red arrowheads point to the absence of venous outflow with grade 0.

As shown in Figure 1, the CCC model evaluated collateral status across three components: pial arterial collaterals (PACs), tissue-level collaterals (TLCs), and venous outflow (VO) profiles (Faizy et al., 2022). PACs were graded on CTA using the Tan scale. The hypoperfusion intensity ratio (HIR) (the volume of tissue with Tmax >10 s divided by the volume of Tmax >6 s) defines HIR ≤ 0.4 as favorable TLCs. The cortical vein opacification score (COVES) on single-phase CTA was used to quantify VO profiles, which were categorized as favorable (COVES 3–6) or unfavorable (COVES 0–2) by assessing the opacification of the vein of Labbé, sphenoparietal sinus, and superficial middle cerebral vein (range 0–6). Two blinded neuroradiologists independently assessed all Tan scale and COVES measurements. Discrepancies were resolved by adjudication by a third neuroradiologist. All raters remained blinded to clinical outcomes during analysis. Patients demonstrating a favorable CCC profile (CCC+) concurrently met all three criteria: robust PACs (Tan grade ≥ 2), favorable TLCs (HIR ≤ 0.4), and favorable VO (COVES 3–6).

### 2.3 Endovascular treatment protocol and cerebral circulation time assessment

Eligibility for MT was determined by neurointerventionalists based on the confirmed presence of a target mismatch profile assessed via perfusion imaging. Pre-thrombectomy diagnostic DSA was systematically performed to evaluate the occlusion site and collaterals as a basis for endovascular strategy. Collateral flow grading was estimated through the American Society of Interventional and Therapeutic Neuroradiology/ Society of Interventional Radiology (ASITN/SIR) system. Successful recanalization was defined by mTICI scores of 2b-3. Bailout angioplasty or stenting (BAOS) was considered for patients failing to achieve recanalization (mTICI 0–2a) or those with persistent, hemodynamically significant residual stenosis (>70%) after 1–3 thrombectomy attempts (stent-retriever, contact aspiration, or combined technique), provided the occluded segment was deemed amenable to rescue angioplasty/stenting by the treating neurointerventionalist (Gao et al., 2024). All subjects were examined using a PHILIPS angiography unit (UNIQ Clarity FD20, The Netherlands) with a power injector (4 mL of Iodixanol injection at 6 mL/s, 200 psi). The imaging parameters were four frames per second for the arterial-to-capillary phase and two frames per second for the venous phase. The location of the catheter during all EVT procedures was determined by the interventionalist.

**Figure 2:**
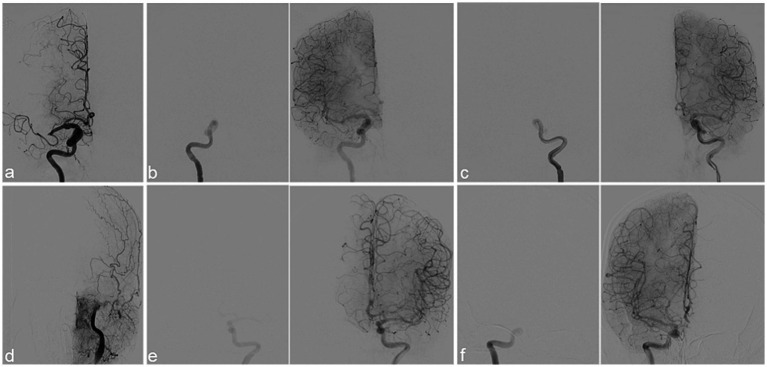
Schematic diagram of the measurement of cerebral circulation time (CCT). **(a)** Angiography suggested right M1 middle cerebral artery occlusion; **(b)** after successful recanalization, the appearance of the siphon segment of the right internal carotid artery was shown as frame 7 of sequence 10; the opacification of the arterial terminal segment was shown as frame 17 of sequence 10; CCT of the stroke side (sCCT) is calculated as (17–7)/4 = 2.5 s; **(c)** the appearance of the siphon segment of the left internal carotid artery was shown as frame 7 of sequence 6; the opacification of the arterial terminal segment was shown as frame 21 of sequence 6; CCT of the contralateral healthy side (hCCT) is calculated as (21–7)/4 = 3.5 s; the relative change of sCCT vs. hCCT (cCCT) is calculated as (2.5–3.5)/3.5 = −0.29. **(d)** Angiography suggested left internal carotid artery occlusion; **(e)** after successful recanalization, the appearance of the siphon segment of the left internal carotid artery was shown as frame 7 of sequence 15; after successful recanalization, the opacification of the arterial terminal segment in the left side was shown as frame 18 of sequence 10; CCT of the stroke side (sCCT) is calculated as (18–7)/4 = 2.75 s; **(f)** the appearance of the siphon segment of right internal carotid artery was shown as frame 9 of sequence 4; the opacification of the arterial terminal segment was shown as frame 18 of sequence 4; CCT of the contralateral healthy side (hCCT) is calculated as (18–9)/4 = 2.25 s; the relative change of sCCT vs. hCCT (cCCT) is calculated as (2.75–2.25)/2.25 = 0.22.

As shown in Figure 2, CCT was measured as the interval between the opacification of the intradural segment of the internal carotid artery and the end of the arterial phase. Two CCT measurements were derived: (1) healthy side CCT (hCCT), based on DSA performed on the normal side before EVT and (2) stroke side CCT (sCCT), based on post-recanalization DSA. The relative change in CCT (cCCT) was computed as cCCT = (sCCT-hCCT)/hCCT. Two specialized evaluators, blinded to clinical data, independently interpreted all DSA sequences, with the mean value utilized for analysis to reduce error in data acquisition.

### 2.4 Outcome measurement

All the included subjects were categorized into two groups based on 3-month functional outcomes. The FR group included all patients with a 3-month mRS score >3, whereas the ER group included those achieving a 3-month mRS score ≤3.

### 2.5 Statistical analysis

Statistical analyses were executed using SPSS 22.0 (IBM Corporation, Armonk, New York, USA) and R software (version 4.2.1). A clinical comparison was performed between the FR and ER cohorts. Normally distributed continuous variables were assessed via the *t*-test, with the results displayed as mean and standard deviation (SD). Non-normally distributed data were analyzed using non-parametric tests and are presented as median and interquartile range (IQR). Categorical variables were compared using Pearson’s chi-squared or Fisher’s exact tests as appropriate. All analyses used two-tailed testing, with statistical significance defined as a *p*-value of <0.05. Intraclass correlation coefficient (ICC) with 95% confidence intervals (CI) were calculated to assess the interobserver reliability of cCCT measurements (two-way random model; absolute agreement conservative type) between two evaluators.

### 2.6 Construction and evaluation of models

Missing data were examined and imputed using multiple imputation using the “mice” package in R, with the number of multiple imputations set to five. The variable “ischemic core volume” and “Tmax” had the highest proportion of missingness (7.14%), followed by “CCC” (6.59%), “cCCT” (4.40%), and “ASPECTS” (4.40%). Specifically, predictive mean matching (PMM) was used for continuous variables, and logistic regression (logreg) was applied to binary categorical variables. To minimize overfitting or skewed indicator distributions, the least absolute shrinkage and selection operator (LASSO) regression with a binomial family was used for feature selection. The optimal penalty parameter (*λ*) was optimized via a 10-fold cross-validation procedure to mitigate overfitting. Multicollinearity among candidate variables was assessed using variance inflation factors (VIFs). Variables retaining non-zero coefficients in the LASSO model were entered into multivariate logistic regression (*p* < 0.05) to identify potential independent predictors. The resulting significant indicators were used to construct the prognostic nomogram. Each predictor was assigned a weighted point score based on its regression coefficient. A summation of these points generated an aggregate risk score, which was subsequently mapped to FR probability via a transformation function. Finally, a conventional nomogram was constructed using the “rms” package in R.

Several verification techniques were used to assess the model’s performance. The area under the receiver operating characteristic curve (AUC) quantified predictive accuracy and identified optimal probability thresholds distinguishing the ER and FR cohorts. Agreement between predicted probabilities and observed outcomes was evaluated via calibration curves. To account for overfitting and obtain unbiased performance estimates, the optimism-corrected values for the AUC, Brier score, and calibration slope and intercept were calculated with bootstrap internal validation with 1,000 resamples (*B* = 1,000). Decision curve analysis (DCA) estimated the net benefit across the full range of threshold probabilities to evaluate clinical applicability.

## 3 Results

### 3.1 Patient characteristics

Of the 332 AIS-LVO patients who underwent EVT during the study period, we finally included 182 patients (Supplementary Figure 1). The mean age was 67.15 ± 12.47 years, with males accounting for 58.79%. On admission, severe stroke (NIHSS ≥15) was seen in 46.15% of patients, and 6.25% had a large ischemic core (ASPECT <6). According to the pre-operative CTA, 29 patients had a favorable CCC. The most common etiology was CE (60.99%), with M1 being the most easily affected location (71.98%). During the EVT procedure, BAOS was performed in 30 cases (8.79% of cases), with 8.79% were angioplasty only. Complete recanalization (mTICI grade 3) was achieved in 75.82% of patients, with a median time of 407.50 min from symptom onset. Successful recanalization was accomplished in 100 cases after the first pass. The median cCCT was 0.00 (−0.17–0.11). Intraclass correlation analyses revealed good consistency between interobservers (ICC = 0.804; 95% CI, 0.570–0.912; *p* < 0.001). HT was found in 57 patients (31.32%) (Table 1).

**Table 1:** Baseline characteristics of included patients.

Variables	Overall (*n* = 182)	FR (*n* = 73)	ER (*n* = 109)	*p*-value
Clinical characters				
Age, y	67.15 ± 12.47	68.86 ± 12.13	66.01 ± 12.62	0.131
Age > 65, *n* (%)	118 (64.84%)	52 (71.23%)	66 (60.55%)	0.156
Male, *n* (%)	107 (58.79)	39 (53.42)	68 (62.39)	0.282
Comorbidities, *n* (%)				0.352
Hypertension	126 (69.23)	54 (73.97)	72 (66.06)	
Diabetes	40 (21.98)	22 (30.14)	18 (16.51)	
CAD	43 (23.63)	12 (16.44)	31 (29.25)	
Previous stroke	51 (28.02)	23 (31.51)	28 (25.69)	
Others	33 (18.13)	16 (21.92)	17 (15.60)	
Symptom, *n* (%)				0.406
Limb weakness	146 (80.22)	51 (69.86)	95 (87.16)	
Hypoesthesia	31 (17.03)	13 (17.81)	18 (16.51)	
Aphasia	90 (49.45)	34 (46.58)	56 (51.38)	
Disturbance of consciousness	15 (8.24)	9 (12.33)	6 (5.50)	
Etiology, *n* (%)				
CE	111 (60.99)	48 (65.75)	63 (57.80)	0.352
LAA	63 (34.62)	21 (28.77)	42 (38.53)	
OE	8 (4.40)	4 (5.48)	4 (3.67)	
NIHSS on admission	14.00 (10.00–18.00)	15.00 (11.00–20.00)	13.00 (9.50–17.00)	0.005^**^
Severe stroke, *n* (%)	84 (46.15)	40 (54.79)	44 (40.37)	0.069
Radiological characters				
Location, *n* (%)				0.685
ICA	43 (23.63)	18 (24.66)	25 (22.94)	
MCA	131 (71.98)	53 (72.60)	78 (71.56)	
ACA	8 (4.40)	2 (2.74)	6 (5.50)	
ASPECT	8.00 (6.00–9.00)	7.00 (6.00–8.00)	8.00 (7.00–9.00)	0.058
ASPECT ≤ 7, *n* (%)	63 (34.62)	27 (36.99)	36 (33.03)	0.405
ASPECT < 6, *n* (%)	10 (6.25)	5 (8.20)	5 (5.05)	0.507
Ischemic core, mL	11.15 (1.85–35.22)	17.39 (3.70–53.50)	6.70 (1.30–29.00)	0.029^*^
Penumbra, mL	102.50 (70.20–144.10)	103.00 (70.20–142.70)	98.90 (70.03–147.98)	0.786
HIR	0.13 (0.01–0.37)	0.12 (0.00–0.41)	0.13 (0.02–0.32)	0.741
HIR > 0.4	27 (14.84)	13 (17.81)	14 (12.84)	0.190
ASITN/SIR	2.00 (1.00–2.00)	1.00 (1.00–2.00)	2.00 (1.00–2.00)	0.014^*^
0–2, *n* (%)	138 (82.63)	61 (91.04)	77 (77.00)	0.022^*^
Tan	2.00 (1.00–2.00)	1.00 (1.00–2.00)	2.00 (1.00–2.00)	0.046^*^
0–1, *n* (%)	69 (37.91)	33 (45.21)	36 (33.03)	0.049^*^
COVES	2.00 (2.00–3.00)	2.00 (1.00–4.00)	2.00 (2.00–3.00)	0.352
0–2, *n* (%)	79 (55.24)	34 (60.71)	45 (51.72)	0.307
CCC	1.00 (0.00–1.00)	1.00 (1.00–1.00)	1.00 (0.00–2.00)	0.367
Favorable CCC	29 (15.93)	6 (8.22)	23 (21.10)	0.045^*^
cCCT	0.00 (−0.17–0.11)	0.04 (−0.08–0.20)	−0.07 (−0.22–0.05)	0.006^**^
Procedural characters				
IVT, *n* (%)	56 (30.77)	21 (28.77)	35 (32.11)	0.743
ORT, min	407.50 (308.50–558.50)	400.00 (312.50–526.50)	419.00 (294.00–588.00)	0.474
PRT, min	61.00 (45.00–86.50)	68.00 (50.00–89.00)	56.00 (42.00–83.50)	0.041
Passes	1.00 (1.00–2.00)	2.00 (1.00–3.00)	1.00 (1.00–2.00)	0.003^**^
First-pass, *n* (%)	100 (54.95)	32 (43.84)	68 (62.39)	0.016^*^
Passes > 3, *n* (%)	6 (3.30)	3 (4.11)	3 (2.75)	0.685
BAOS	30 (16.48)	12 (16.44)	18 (16.51)	>0.999
Angioplasty	16 (8.79)	7 (9.59)	9 (8.26)	
Stenting	14 (7.69)	5 (6.85)	9 (8.26)	
mTICI, *n* (%)				0.724
2b	44 (24.18)	19 (26.03)	25 (22.94)	
3	138 (75.82)	54 (73.97)	84 (77.06)	
HT, *n* (%)	57 (31.32)	33 (45.21)	24 (22.02)	0.001^**^
Laboratory characters				
HS-CRP, mg/L	3.61 (1.33–9.40)	4.49 (1.24–12.92)	2.87 (1.33–8.35)	0.279
HS-CRP>3, *n* (%)	96 (52.75)	44 (60.27)	52 (47.71)	0.130
Creatine, μmol/L	69.50 (57.75–89.00)	72.00 (54.50–95.50)	69.00 (59.00–85.50)	0.748
Glucose, mmol/L	7.00 (5.95–8.69)	7.69 (6.43–11.03)	6.55 (5.64–8.00)	<0.001^***^
Glucose > 6.1	129 (70.88)	59 (80.82)	70 (64.22)	0.020^*^
Fbg, g/L	3.10 (2.68–3.70)	3.10 (2.71–3.90)	3.10 (2.60–3.64)	0.307
Fbg > 3.8	41 (22.53)	20 (27.40)	21 (19.27)	0.210
Neutrophils, *10^9^/L	5.11 (3.77–7.02)	5.50 (3.72–7.62)	4.93 (3.79–6.73)	0.384
Lymphocytes, *10^9^/L	1.61 (1.07–2.52)	1.35 (0.97–2.45)	1.71 (1.20–2.53)	0.068
Monocytes, *10^9^/L	0.41 (0.29–0.60)	0.43 (0.30–0.63)	0.41 (0.29–0.58)	0.642
SIRI, *10^9^/L	1.26 (0.70–2.15)	1.48 (0.69–2.49)	1.17 (0.70–1.86)	0.112
NLR	2.97 (1.74–5.54)	3.78 (1.68–7.39)	2.80 (1.80–4.74)	0.152
NLR > 3.75, *n* (%)	73 (40.11)	37 (50.68)	36 (33.03)	0.021^*^

FR, futile recanalization; ER, effective recanalization; CAD, coronary artery disease; CS, cardiogenic stroke; LAA, large artery atherosclerosis; OE, other etiology; NIHSS, National Institutes of Health Stroke Scale; ICA, internal carotid artery; MCA, middle cerebral artery; ACA, anterior cerebral artery; ASPECT, Alberta Stroke Program Early CT score; HIR, hypoperfusion intensity ratio; ASITN/SIR, American Society of Interventional and Therapeutic Neuroradiology/Society of Interventional Radiology scale; CTA-ASPECT, Alberta Stroke Program Early Computed Tomography Score; COVES, Cortical Vein Opacification Score; CCC, cerebral collateral cascade; cCCT, relative change of cerebral circulation time; IVT, intravenous thrombolysis; ORT, onset to reperfusion time; BAOS, bailout intracranial angioplasty or stenting; mTICI, modified Thrombolysis in Cerebral Infarction Score; HT, hemorhagic transformation; HS-CRP, hypersensitive C reactive protein; Fbg, fibrinogen; SIRI, Systemic inflammatory response index; NLR, neutrophil-to-lymphocyte ratio.

^*^0.01 < *p*-value <0.05; ^**^0.001 < *p*-value <0.01; ^***^p-value <0.001.

### 3.2 Comparison between FR and ER groups

Favorable functional outcome (59.89%) was reported in 109 patients after 90 days and was found to have better NIHSS (13.00 [9.50–17.00] vs. 15.00 [11.00–20.00], *p* = 0.005), lower baseline ischemic core volume (6.70 [1.30–29.00]ml vs. 17.30 [3.70–53.50]ml, *p* = 0.029), better arterial collateral status (ASITN/SIR, 2.00 (1.00–2.00) vs. 1.00 [1.00–2.00], *p* = 0.014; Tan, 2.00 [1.00–2.00] vs. 1.00 [1.00–2.00], *p* = 0.046) and lower cCCT (−0.07 [−0.22–0.05] vs. 0.04 [−0.08–0.20], *p* = 0.006). Additionally, the FR group had fewer patients with favorable CCC (8.22% vs. 21.10%, *p* = 0.045) and more patients with higher glucose level>6.1 mmol/L (80.82% vs. 64.22%, *p* = 0.020) and higher NLR>3.75 (50.68% vs. 33.03%, *p* = 0.021) according to the pre-operative imaging and laboratory results. During the EVT procedure, the ER cohort had a shorter delay from groin puncture to the final mTICI score (56.00 [42.00–83.50] min vs. 68.00 [50.00–89.00] min in the FR cohort, *p* = 0.041), and fewer attempts were performed to achieve successful recanalization in the ER group (1.00 [1.00–2.00] vs. 2.00 [1.00–3.00], *p* = 0.003) with 62.39% having a first-pass effect (vs. 43.84% in the FR cohort, *p* = 0.016). On postinterventional CT, more patients in the FR group suffered from HT (45.21% vs. 22.02%, *p* = 0.001) (Table 1).

### 3.3 Derivation of the prediction model

**Figure 3:**
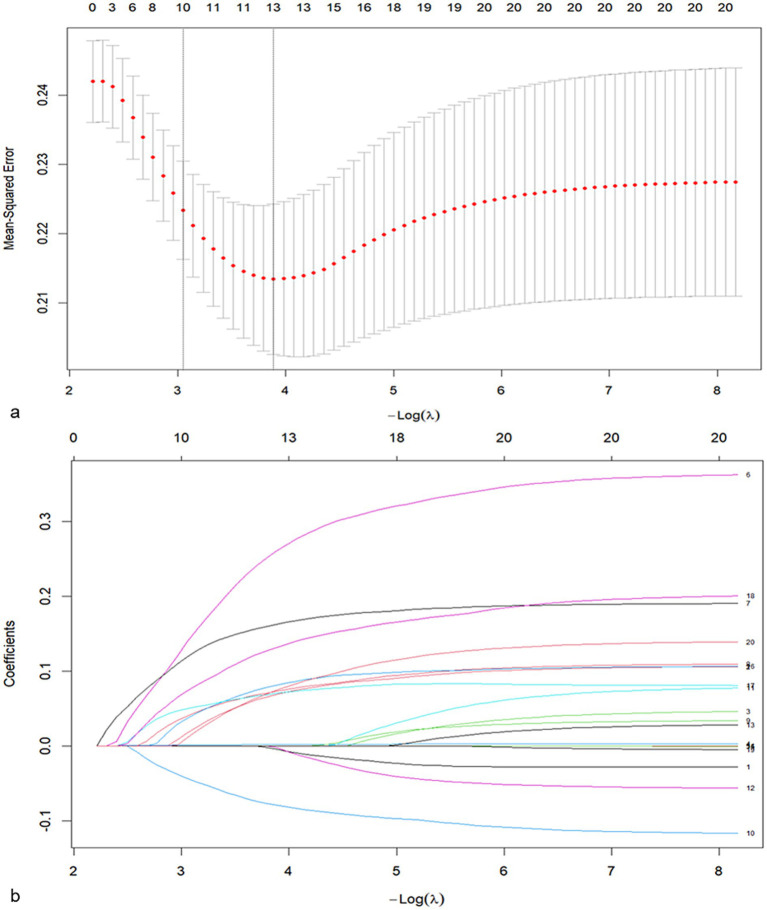
Feature selection using LASSO (least absolute shrinkage and selection operator) regression. **(a)** LASSO coefficient profiles of 20 risk factors. Each plot was constructed against the log (lambda) sequence. Thirteen variables with non-zero coefficients were selected by minimum lambda. **(b)** LASSO regression cross-validation curve. The two dotted vertical lines were drawn at the optimal scores by the minimum criteria and one standard error criteria.

A total of 20 baseline and intra-interventional variables were introduced into the LASSO regression model. Thirteen variables, including male, age>65 years old, ischemic core volume, cCCT, CCC, baseline severe stroke, first-pass recanalization, mTICI3, ORT, high HS-CRP level >3.0 mg/L, high glucose level >6.10 mmol/L, high NLRs >3.75, and high fibrinogen level >3.8 g/L were selected and entered with non-zero coefficients in the LASSO regression model using lambda. Min = 0.020 (Figure 3). With the variance inflation factor (VIF) values being < 2, no obvious multicollinearity existed for the significant variables that were entered into multivariate analysis (Supplementary Figure 2).

**Figure 4:**
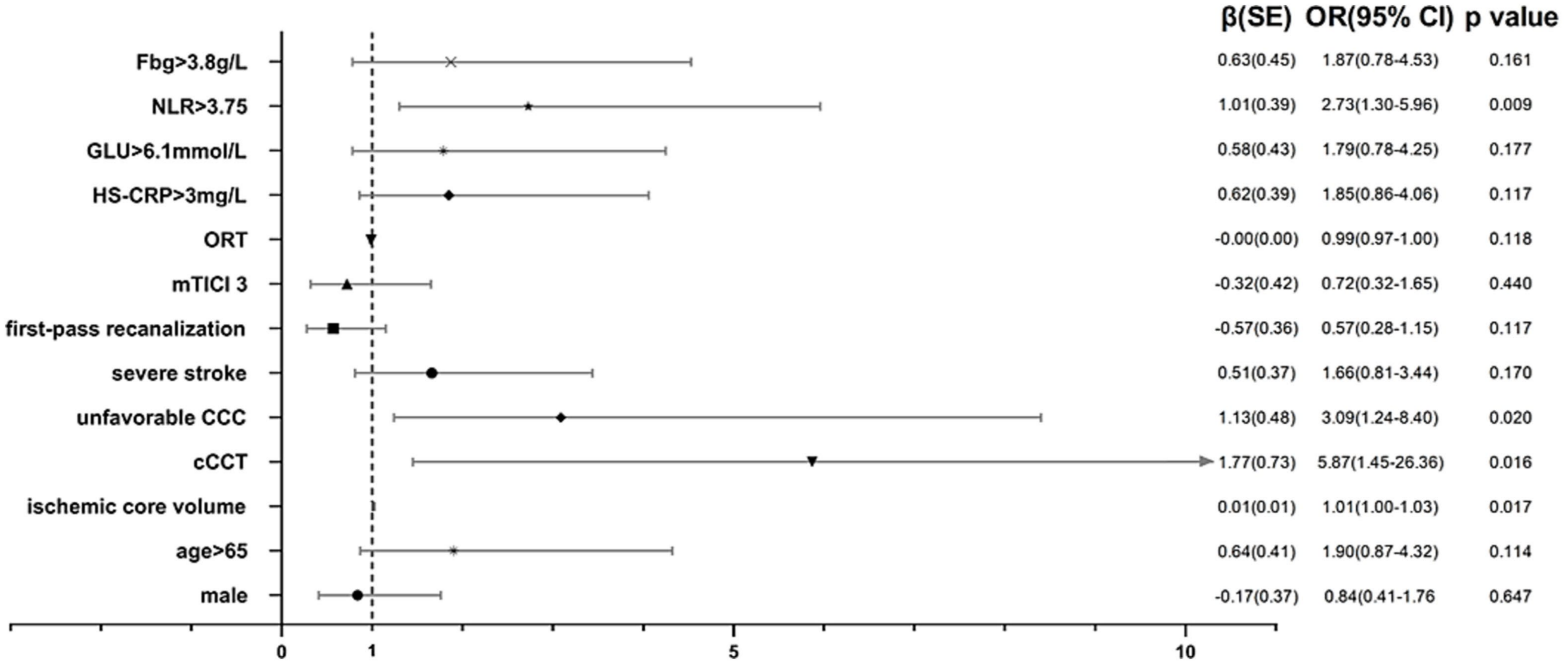
Multivariable logistic regression analyses. cCCT, relative change of cerebral circulation time; CCC, cerebral collateral cascade; mTICI, modified thrombolysis in cerebral infarction; ORT, onset to reperfusion time; HS-CRP, high sensitivity C-reactive protein; GLU, glucose; NLRs, neutrophil-to-lymphocyte ratios; Fbg, fibrinogen; CI, confidence interval.

**Figure 5:**
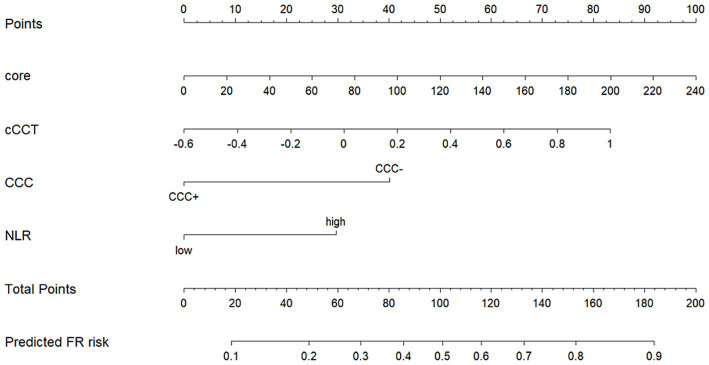
Comprehensive model exhibited by a nomogram. Core, ischemic core volume; cCCT, relative change of cerebral circulation time; CCC, cerebral collateral cascade; NLR, neutrophil-to-lymphocyte ratio.

In the multivariate analysis, odds ratios (ORs) and *p*-values with 95% CIs were calculated. Unfavorable CCC status (OR, 3.09 [95% CI, 1.24–8.40], *p* = 0.020), cCCT (OR, 5.87 [95% CI, 1.45–26.36], *p* = 0.016), ischemic core volume (OR, 1.01 [95% CI, 1.00–1.03], *p* = 0.017), and high NLRs >3.75 (OR, 2.73 [95% CI, 1.30–5.96], *p* = 0.009) were independent predictors of FR. The multivariable predictors are listed in Figure 4. The final individualized prediction model for FR was visualized using a nomogram (Figure 5). For example, the nomogram assigned a > 70% probability of adverse consequence in a stroke patient with an ischemic core volume of 60 mL (25 points), cCCT of 0.2 (41 points), unfavorable CCC (40 points), and high-level NLRs of 5.230 (30 points) with a total score of 136 points. On the other hand, a < 10% probability of unfavorable outcome was nominated for another patient, with an ischemic core volume of 20 mL (8 points), cCCT of −0.4 (10 points), favorable CCC (0 point), and a low-level NLR of 1.869 (0 point), with a total score of 18 points.

### 3.4 Validation of the prediction model

**Figure 6:**
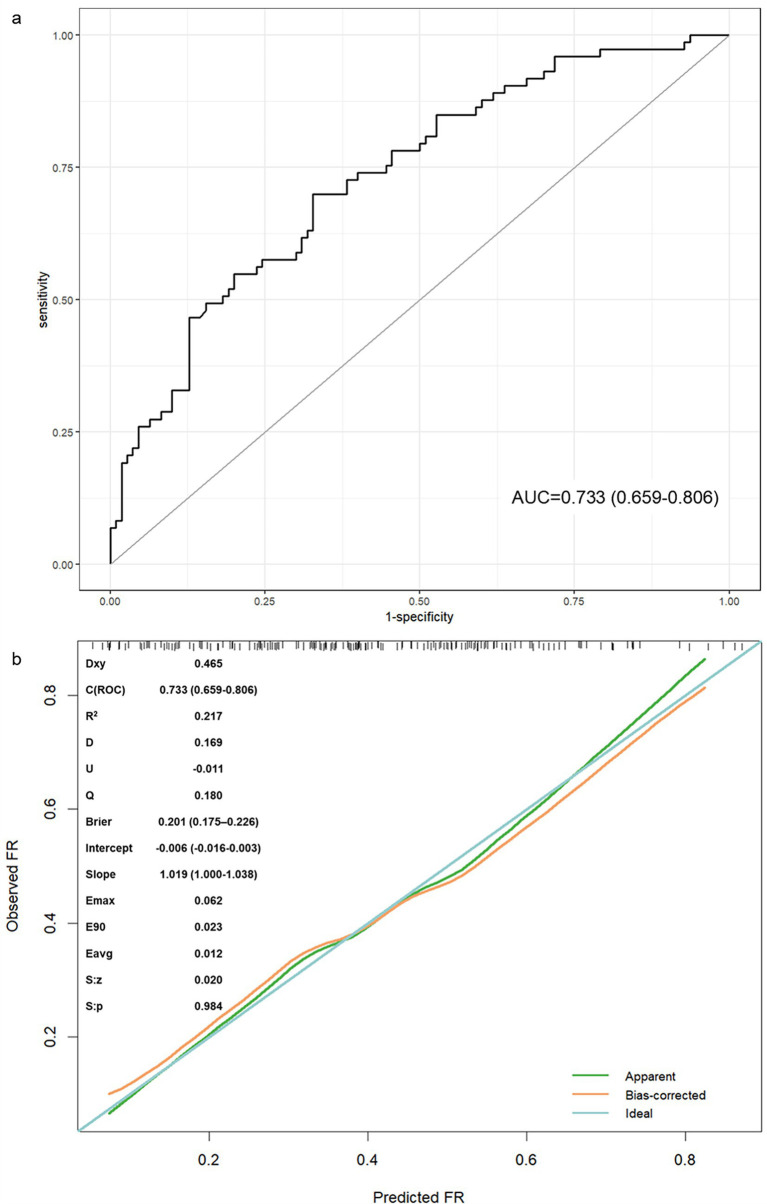
Receiver operating characteristic (ROC) curve analysis and calibration curve of the nomogram model. **(a)** ROC curve showing an AUC (area under the curve) value of 0.733; **(b)** calibration curve.

**Figure 7:**
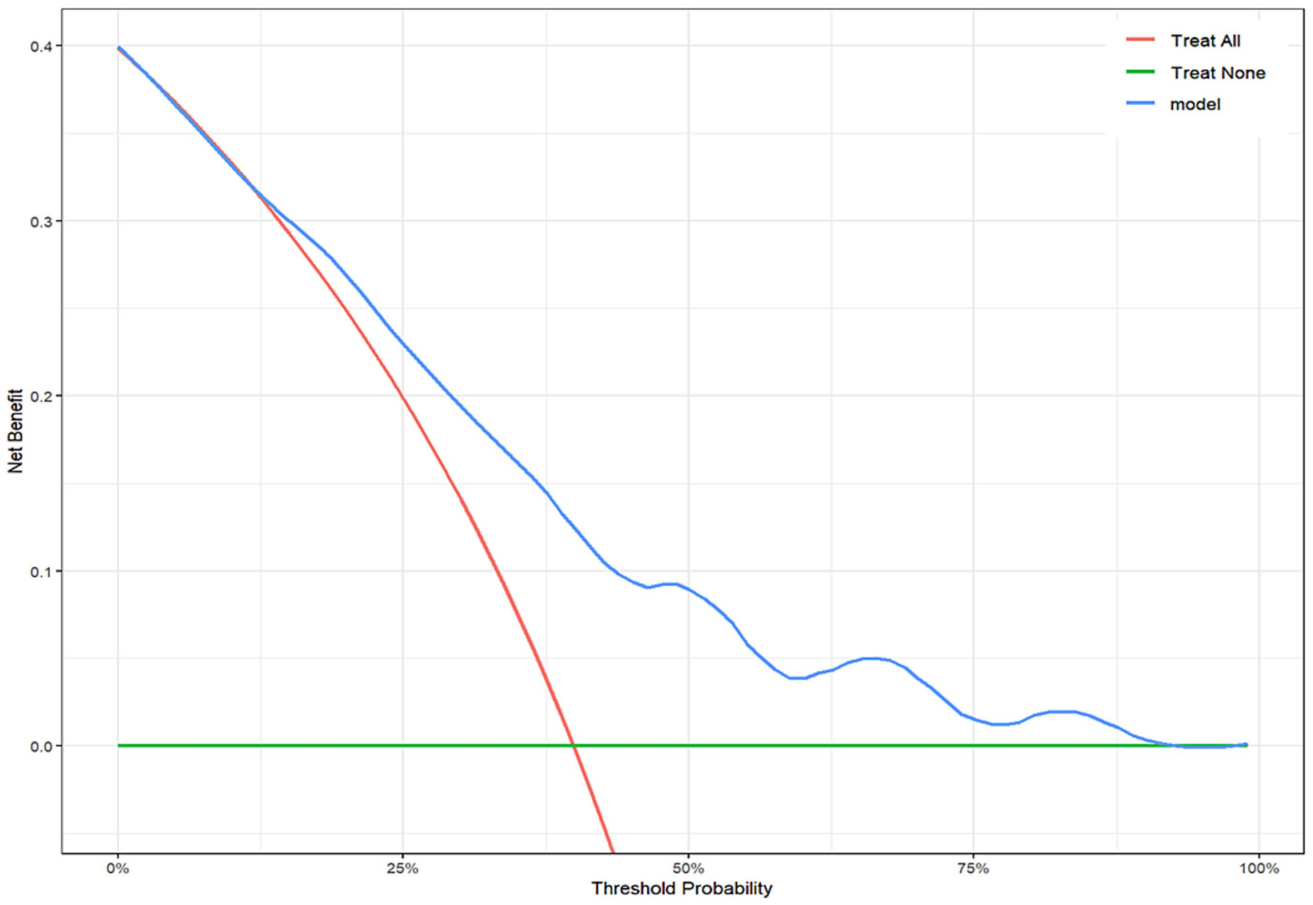
Decision curve analysis (DCA) of the nomogram model for determining futile recanalization.

Internal validation used 1,000 bootstrap resamples. The model allowed integration of baseline and intra-interventional characteristics and exhibited strong discriminatory power with an AUC-ROC value of 0.733 (95% CI, 0.659–0.806) (Figure 6). The calibration curve revealed a well-calibrated slope of 1.019 (intercept: −0.006) and confirmed reliable predictive accuracy (Brier, 0.201, [95% CI, 0.175–0.226]). Non-significant Hosmer–Lemeshow test results (*p* = 0.770) indicated appropriate model calibration (Figure 7). The DCA curve demonstrated a relatively favorable net clinical benefit at threshold probabilities of 10 to 90% (Figure 7).

## 4 Discussion

This study represented the first integrated assessment of the association between microcirculatory perfusion and hemodynamics (CCC and cCCT) and plasma biomarkers potentially reflecting systematic inflammatory profile and no-reflow (NLR) with FR after EVT in AIS-LVO patients, and allowed comprehensive modeling of baseline and intra-procedural characteristics for predicting FR after successful mechanical recanalization. The new comprehensive risk score showed improved predictive accuracy (AUC, 0.733 [0.659–0.806]) and calibration (Brier, 0.201 [0.175–0.226]; Hosmer–Lemeshow test, *p* = 0.770). The nomogram developed in this study provided a practical tool for predicting FR following EVT. By providing a quantifiable risk estimate and stratification, this approach might assist clinicians in early identification of patients at high risk for FR, allowing for prioritized intensive monitoring and individualized post-thrombectomy management. For instance, patients identified as high-risk by the model could be prioritized for intensified hemodynamic monitoring or targeted adjunctive therapies in a neurocritical care setting, potentially optimizing resource use. More broadly, this model underscored a paradigm shift—from the traditional “time window” concept to a more refined “tissue window” approach and from solely pursuing successful macrovascular recanalization to actively assessing and addressing microcirculatory failure. The incorporation of parameters such as CCT and CCC reinforced the critical role of cerebral microcirculatory dysfunction (no-reflow) in FR, shifting the therapeutic focus beyond macrovascular reperfusion and highlighting specific pathophysiological targets for future research targeting microcirculation protection in high-risk patients.

The pathophysiology underlying FR remains incompletely characterized and involves multifactorial mechanisms. Key contributors include microvascular dysfunction (e.g., no-reflow phenomenon), early arterial reocclusion, hemorrhagic transformation, and inadequate collateral circulation (Tian et al., 2023; Deng et al., 2022). No-reflow (microvascular failure) (Taskiran-Sag et al., 2018; Ng et al., 2022; Rezkalla and Ra, 2002), together with early arterial reocclusion and impaired cerebral autoregulation, predominantly occur within the first 24-h post-recanalization (Schiphorst et al., 2020; Abbasi-Habashi et al., 2025), suggesting that FR is driven by acute mechanisms. However, the widely adopted definition of FR in real-world clinical trials is failure to achieve a favorable functional outcome (mRS 3–6 or 4–6) at 90 days despite successful recanalization. With increasing evidence that minimal assistance required (mRS 3) may still have a good quality of life and patients who achieve mRS 3 more closely resemble mRS 2 than mRS 4 patients based on functional disability (Hong and Saver, 2009) and quality of life (Rangaraju et al., 2017), as well as long-term survival (Magalhães et al., 2014), it is more reasonable to utilize mRS 4–6 as a measure of poor/futile outcome (Dhillon et al., 2023; Gan et al., 2026; Meinel et al., 2020). This 90-day endpoint captures the ultimate patient-centered outcome, integrating both early pathophysiological events and subsequent recovery processes. Our predictive model, by incorporating microvascular metrics (cCCT and CCC) and the inflammation parameter (NLR), targets the very early phase of this cascade characterized by neuroinflammation, oxidative stress, and microvascular failure (Elmadhoun et al., 2024; Li Z. et al., 2025). Reconciling acute pathophysiology with the late outcome measure could facilitate early identification of the high-risk FR population at the pre- and intra-EVT stages to better inform shared decision-making and improve personalized treatment strategies, including intensive monitoring or the use of adjuvant treatment.

Representing the transit duration of blood flow through brain parenchyma, prolonged CCT may reflect the poor pathophysiology of the ischemic penumbra, damaged arterial inflow, and compromised venous drainage after EVT, signifying increased resistance and impaired autoregulatory vasodilation of small vessels and a compromised cerebral microcirculatory state (Li S. et al., 2025; Li Z. et al., 2025; Wang J. Q. et al., 2022; Gölitz et al., 2017; Wang Y. J. et al., 2022). Being able to reflect microcirculation obstruction and microvasculature disturbance, CCT would be a promising and readily available cerebral hemodynamic parameter, facilitating immediate identification of no-reflow susceptibility during recanalization procedures (Sperring et al., 2023), which is widely recognized as one of the most critical pathophysiological mechanisms underlying FR. In this study, DSA-derived CCT was used to evaluate its prognostic utility in AIS-LVO patients. We demonstrated that post-EVT cCCT was an independent and strong predictor of clinical functional outcome, which is in accordance with previous studies (Wang J. Q. et al., 2022; Wang Y. J. et al., 2022).

Collateral circulation status is an established prognostic indicator following reperfusion therapy for AIS (Leng et al., 2016; van den Wijngaard et al., 2015). As a comprehensive collateral factor involving all the anatomic and functional compartments of brain vasculature—arteries (arterial collaterals), capillaries (tissue-level perfusion), and veins (venous outflow profiles)—CCC was established as a surrogate indicator of post-ischemic microvascular integrity (Faizy et al., 2022) and tissue perfusion deficits (Nagakane et al., 2012). Compared to arterial/venous-only collateral metrics, favorable CCC profiles demonstrated better neuroprotective effects by more effectively attenuating brain tissue injury, minimizing ischemic core formation (Huang et al., 2025), and mitigating brain edema development (Faizy et al., 2024), thus facilitating better functional outcomes in EVT-treated stroke patients. In this study, our analysis identified unfavorable CCC profiles as an independent determinant of FR after EVT, catalyzing a transition from the temporally constrained “time window” criteria toward individualized tissue viability assessment in the management of LVO-AIS.

In recent years, accumulating clinical evidence implicates inflammation in ischemic stroke pathophysiology and prognosis prediction (Guo et al., 2024). Post-ischemic cellular damage initiates a neuroinflammation cascade characterized by the release of inflammatory mediators (Macrez et al., 2011). Concurrently, peripheral leukocytes compromise blood–brain barrier integrity, inducing secondary neuronal damage and exacerbating neurological deficits (Yang et al., 2022; Westendorp et al., 2022). Multiple inflammation-related serological biomarkers demonstrate independent prognostic value in AIS, especially the novel composite parameter such as NLRs (Wang et al., 2024; Gong et al., 2021; Chen et al., 2024; Cong et al., 2024), which is further corroborated by our investigation. As an integrative inflammation biomarker synthesizing various inflammatory parameters, NLRs provide enhanced immunological profiling. This comprehensive representation partially explains its predictive utility for FR pathogenesis and progression. Emerging evidence indicates alternative pathways may mediate the association between NLRs and FR. Post-ischemia, neutrophils appear to accumulate in the capillaries, arresting blood flow and aggravating tissue damage despite macrovascular recanalization (Erdener et al., 2020; El Amki et al., 2020; Sun et al., 2024). The neutrophil-mediated capillary stalls contribute to secondary infarct growth by causing distal microvascular disturbances beyond the primary infarct core, establishing NLRs as a diagnostic indicator for no-reflow (Sperring et al., 2023). This may further explain the good predictive performance of NLRs in our model.

Several predictive algorithms have been developed to predict the outcome of individual patients treated with EVT. However, no single model has demonstrated optimal utility for patient selection nor achieved widespread clinical practice (Kremers et al., 2022). Existing frameworks, including MR PREDICTS (Venema et al., 2017; Sarraj et al., 2013), PRE (Rangaraju et al., 2015), and the new ACT-BAN Scale, share some limitations. First, they were primarily designed to predict general functional outcomes, irrespective of recanalization success, rather than to specifically address FR prognostication. Second, their predictor sets were largely confined to conventional clinical and imaging factors such as age, NIHSS, ASPECTS, and glucose. Investigations dedicated to predicting FR in AIS-LVO patients post-EVT remain scarce (Gong et al., 2025; Huang et al., 2024; Guan et al., 2023). To address this gap, our research pioneers the integration of multidimensional pre- and intra-procedural parameters, including comprehensive information on traditional clinical, laboratory, imaging, and workflow variables alongside novel microvascular perfusion metrics and collateral circulation dynamics into the establishment of the prediction model. Notably, our nomogram uniquely integrated cCCT dynamics with CCC profiles to quantify microcirculatory dysfunction and proved significant predictive value for FR after EVT. However, the model developed in our study showed only moderate AUC, though it had good calibration and favorable clinical net benefit. This may be partly attributed to the limited sample size. We also searched previous studies and found that some established models also had similar performance to our model by ROC analysis such as the THRIVE-EVT (AUC = 0.718) (Flint et al., 2022) and the deep learning model developed by Hilbert (AUC 0.71 [95% CI, 0.62–0.75]) (Hilbert et al., 2019). In addition, a previous study validated 19 models systematically in the MR CLEAN registry. Discriminative performance ranged from 0.61 (SPAN-100) to 0.80 (MR PREDICTS). For models that predicted functional outcome with mRS cut points 0 to 3 (good) or 4 to 6 (poor), DRAGON (0.73 [95% CI, 0.71–0.75]), Houston Intra-Arterial Therapy ([HIAT]; 0.71 [95% CI, 0.69–0.73]), HIAT2 (0.69 [95% CI, 0.67–0.70]), mHIAT2 (0.66 [95% CI, 0.64–0.67]), mPRE (0.68 [95% CI, 0.67–0.70]), and mTHRIVE (0.68 [95% CI, 0.66–0.69]) showed similar AUC performance with our model (Kremers et al., 2022). This suggested that despite a modest AUC value, our model demonstrated acceptable discriminatory ability and holds potential clinical utility.

Additionally, there is limited research on the long-term outcomes of EVT. The mortality rate could reach 34.1%, and only 32.9% could achieve functional independence at 12 months or longer after EVT (Wu et al., 2025). A quarter of stroke survivors may have a recurrent stroke at 5 years, and approximately double that at 10 years. Half of the stroke survivors are deceased at 5 years after stroke, and three-fourths at 10 years (Singh et al., 2018). Patients who survive beyond 30 days after a first stroke continue to die at a rate of approximately 10% per year for the next 5 years (Bronnum-Hansen et al., 2001). Therefore, a long-term stroke prognosis based on available AIS explanatory variables also has significant clinical and societal value. Studies have suggested that modifiable risk factors such as smoking, hypertension, and diabetes (Singh et al., 2018; Feigin et al., 2016), as well as nonvascular causes such as economic factors (Delcourt et al., 2011; Lourenço et al., 2021), may play an increasingly important role in this regard. What’s more, a significant proportion of stroke recurrences is prominently influenced by stroke etiology (Lovett et al., 2004; Li et al., 2015). More research is required to investigate newer prognostic factors that affect long-term outcomes for superior stroke management.

Several limitations should be acknowledged when interpreting our findings. First, the measurement of CCT and CCC may be influenced by multiple factors, such as manual quantification and parameter acquisition settings. Second, as a single-center study, all data were derived from patients treated at a tertiary academic center, which limits the generalizability (external validity) of our prediction model to other healthcare settings with different patient mixes. The retrospective design introduces the potential for selection bias and information bias. Given the reliance on the accuracy and completeness of historical medical records, some predictors of interest have missing data, and the influence of unmeasured confounders, such as stroke-related complications, medical management, rehabilitation intensity, and social support, cannot be excluded. Third, the sample size is relatively limited considering the number of candidate predictors initially assessed. Although LASSO regression and rigorous internal validation (bootstrapping) were used to correct for overfitting, the relatively low event-per-variable in our multivariable analysis and the lack of external validation indicate the potential for model overfitting and the importance of representing the broader stroke population. These methodological constraints have direct implications for our model’s performance and clinical applicability. Therefore, external validation in different clinical settings should be emphasized to confirm its generalizability and clinical utility before widespread adoption. Prospective research with a larger sample size should be further conducted to validate our findings.

## 5 Conclusion

A comprehensive nomogram-based prediction model for FR after EVT in AIS-LVO patients is developed and preliminarily validated. Involvement of CCC and CCT improves the evaluation of microcirculatory perfusion and collateral status, thereby enabling this model to be a promising clinical decision tool to provide individualized predictions of the effect of EVT. Despite the favorable discrimination and calibration, the broader applicability of this informative risk prediction model requires additional multicenter research.

## Data Availability

The raw data supporting the conclusions of this article will be made available by the authors, without undue reservation.

## Supplementary material

SUPPLEMENTARY FIGURE 1 Flowchart of subject selection. AIS, acute ischemic stroke; LVO, large vessel occlusion.

SUPPLEMENTARY FIGURE 2 Multicollinearity assessment. VIF, variance inflation factor; cCCT, relative change of cerebral circulation time; CCC, cerebral collateral cascade; ORT, onset to reperfusion time; HS-CRP, high sensitivity C-reactive protein; GLU, glucose; NLR, neutrophil-to-lymphocyte ratio; Fbg, fibrinogen.
