# Supplementary figures and images for: Risk score for futile recanalization: integrating cerebral circulation time and collateral cascade
Source: Front Aging Neurosci. 2026 Apr 10;18:1737846. doi: 10.3389/fnagi.2026.1737846 (PMC13106472; doi:10.3389/fnagi.2026.1737846)

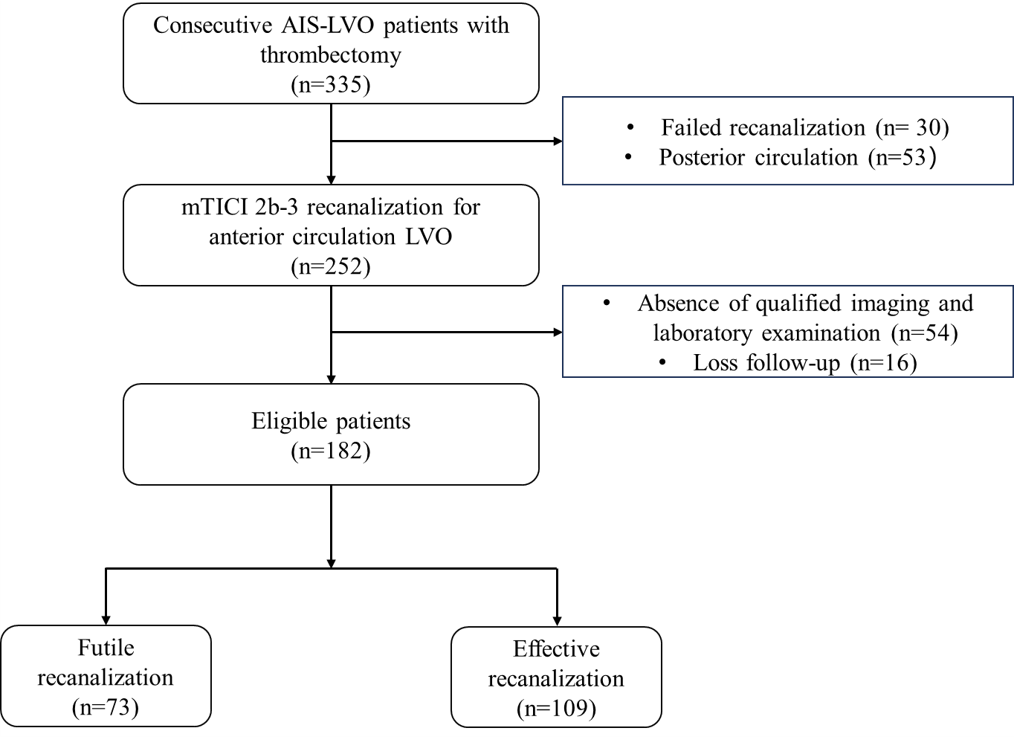

Supplement: SUPPLEMENTARY FIGURE 1 — Flowchart of subject selection. AIS, acute ischemic stroke; LVO, large vessel occlusion. [file Image_1.tif]

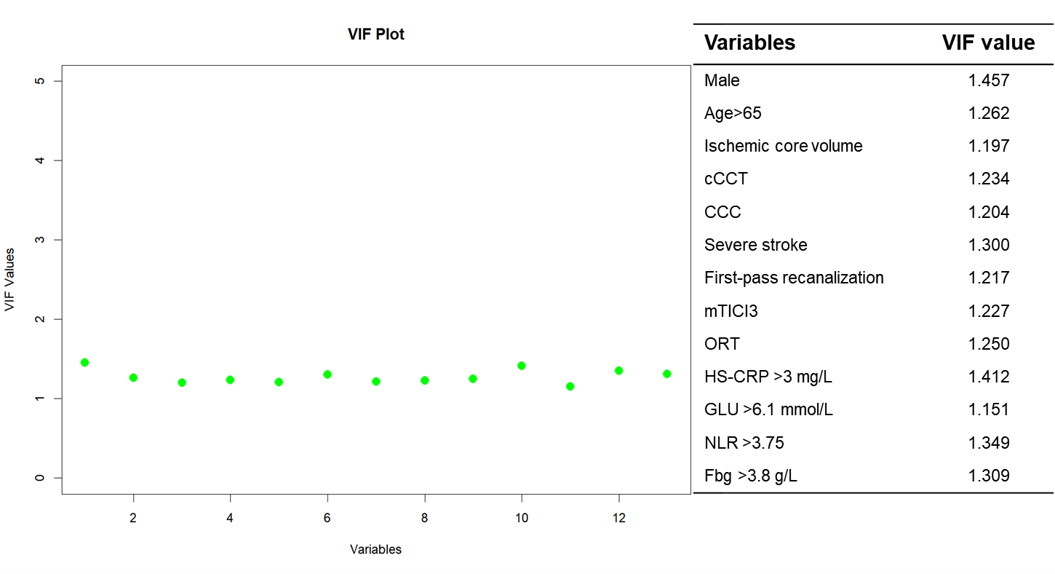

Supplement: SUPPLEMENTARY FIGURE 2 — Multicollinearity assessment. VIF, variance inflation factor; cCCT, relative change of cerebral circulation time; CCC, cerebral collateral cascade; ORT, onset to reperfusion time; HS-CRP, high sensitivity C-reactive protein; GLU, glucose; NLR, neutrophil-to-lymphocyte ratio; Fbg, fibrinogen. [file Image_2.tif]
